# Diagnostic accuracy of SATB2 in identifying primary and metastatic colorectal carcinoma: a comparative immunohistochemical study

**DOI:** 10.3332/ecancer.2022.1491

**Published:** 2022-12-19

**Authors:** Manar S Elnady, Faika A Eltatawy, Azima G Nosseir, Yomna A Zamzam, Dina M El-Guindya

**Affiliations:** Department of Pathology, Faculty of Medicine, Tanta University, Tanta 31527, Egypt; a https://orcid.org/0000-0003-3419-0618

**Keywords:** SATB2, CDX2, colorectal carcinomas (CRCs), metastasis, immunohistochemistry

## Abstract

Special AT-rich sequence-binding protein 2 (SATB2) is a new marker that could identify the colonic origin, but whether its expression is preserved in metastatic colorectal carcinomas (CRCs) remains unclear. This study was designed to investigate SATB2 validity in the identification of CRC either alone or in combination with caudal-type homeobox 2 (CDX2) and/or cytokeratin 20 (CK20). Moreover, we examined the concordance of SATB2 expression in primary CRC and paired metastatic specimen. Immunohistochemical expression of SATB2, CDX2 and CK20 was evaluated in primary CRC, 50 paired metastatic CRC and 80 non-CRC specimens. This study demonstrated that the ideal SATB2 cut-off value for recognising colonic from non-colonic origin was 10%. SATB2 was more sensitive and specific than CK20. However, it was more specific but less sensitive than CDX2. Analysing the combined markers expression, SATB2 and CDX2 combination revealed better sensitivity, specificity and larger area under curve compared to SATB2 alone, CDX2 alone and combined CDX2 and CK20. Moreover, SATB2 was able to retain its expression at the metastatic sites. SATB2 was totally concordant between primary CRC and their paired metastatic sites (concordance rate = 100%) with perfect level of agreement. SATB2 could be considered as an accurate diagnostic marker of primary and metastatic CRC. SATB2 and CDX2 is the best combination serving the highest sensitivity and specificity in detection of CRC.

## Introduction

Colorectal cancer (CRC) is the third most prevalent cancer in both men and women and the fourth most prominent cause of cancer-related deaths worldwide [1]. Pathologic detection of colorectal carcinomas (CRCs) featuring as a colonic mass does not generally pose diagnostic challenges [2]. Metastatic CRCs frequently resemble the histological patterns of adenocarcinomas from other origins; discrimination between these metastases can be problematic particularly in poorly differentiated ones [3]. Diagnostic panels of immunohistochemical (IHC) staining are generally required for the identification of CRC.

It is well-established that CRCs consistently express cytokeratin 20 (CK20) and caudal-type homeobox 2 (CDX2) [4]. However, although CK20 is considered a remarkably sensitive marker, it is also expressed in numerous different types of adenocarcinomas [3]. CDX2 is the most specific existing marker for recognition of the colonic origin. However, it can be also expressed in other tumours like cholangiocarcinoma and ovarian mucinous tumours. Moreover, it was exhibited that poorly differentiated CRCs may lack CDX2 expression [5, 6]. Furthermore, CDX2 loss of expression in liver metastasis was observed in a study by Tóth *et al* [7]. Taken together, there is an unmet requirement for identification of newer combinations of IHC markers with higher sensitivity and specificity in diagnosis of CRC.

The special AT-rich sequence-binding protein 2 (SATB2) is a transcription factor that binds to the nuclear matrix attachment region of DNA and hence regulates transcription and chromatin remodelling [3]. SATB2 was primarily proposed as a new marker of osteoblastic differentiation. It also has a role in central nervous system development showing high levels of expression in the cerebral cortex and the spinal cord [4]. It was demonstrated that the normal lining of the lower gastrointestinal tract exhibited moderate to strong SATB2 expression. Accordingly, it seems to be an upcoming IHC marker for CRC diagnosis. Studies have evaluated SATB2 as a new marker for identification of the colonic origin, their results seem to be promising [4, 8, 9], but whether its expression is preserved in metastatic CRCs remains unclear and warrants further investigations.

This study was designed to investigate the validity of SATB2 in the identification of CRCs either alone or in combination with CK20 and/or CDX2. Moreover, we examined the concordance of SATB2 expression in primary CRC and their paired metastatic CRCs specimens.

## Materials and methods

### Study design and population

This is a retrospective study that was carried out at the Pathology Department, Faculty of Medicine, Tanta University, during the period from April 2019 to December 2021. It included 70 cases of primary CRC as well as 80 cases of non-CRC. In addition, 50 paired metastatic specimens of the primary CRC cases (either nodal or peritoneal deposits) were obtained so as to analyse the concordance of SATB2 expression between primary and metastatic CRCs. The included non-CRC cases exhibited either an intestinal morphology or were poorly differentiated carcinomas. They were incorporated as follows: lung adenocarcinoma (10 cases), oesophageal adenocarcinoma (3 cases), gastric adenocarcinoma (7 cases), small intestine adenocarcinoma (7 cases), pancreatico-biliary carcinoma (8 cases), hepatocellular carcinoma (8 cases), female genital tract adenocarcinoma (19 cases), prostatic adenocarcinoma (6 cases), breast carcinoma (6 cases) and urinary bladder adenocarcinoma (6 cases). The study was approved by the Institutional Research Ethics Committee (reference # 32895).

### Data collection and histopathologic evaluation

For primary CRC cases, clinical data as well as tumour-related features (tumour size, tumour location and gross appearance) were gathered from the accompanying pathology reports and medical records. Primary CRC cases were graded following the 2-tiered grading system into low and high grades, as recommended by the World Health Organization classification [10]. Pathologic staging was determined according to the 8th edition of American Joint Committee on Cancer/Tumour, Node, Metastasis (TNM) staging system [11]. For non-CRC cases, haematoxylin and eosin as well as confirmatory immunohistochemical slides were examined to confirm the diagnosis.

### Tissue microarray (TMA)

For each studied specimen, areas that adequately represent tumour tissues with appropriate preservation were identified. Necrotic areas and those with crushing artefacts were excluded. Tissue microarray (TMA) recipient blocks (6×4 arrays) were produced using the TMA builder mold (CAT# TMA-001, Thermo Fisher Scientific, Runcorn, UK). This is followed by insertion of two tissue cores from areas of interest on paraffin blocks of the studied specimens into the holes on the recipient blocks to form TMA Blocks.

### Immunohistochemical staining

Sections (5 µm thick) mounted on positively charged slides were left to dry for 30 minutes at 37°C. Deparaffinisation and antigen retrieval were carried out using Dako PT Link unit. High pH EnVision^TM^ FLEX Target Retrieval Solutions was used reaching 97°C for 20 minutes. Immunostaining was accomplished using Dako Autostainer Link 48. Antibodies included in this study were SATB2 rabbit monoclonal antibody (clone EPNCIR130A, 1:100 dilution, Abcam, UK), CDX2 mouse monoclonal antibody (Kit no. IS080, ready-to-use, Dako, Glostrup, Denmark) and CK20 rabbit monoclonal antibody (Kit no. M7019, ready-to-use, Dako, Glostrup, Denmark). In brief, slides were kept in Peroxidase-Blocking Reagent for 5 minutes, incubated with primary antibodies for 20–30 minutes, horseradish peroxidase polymer reagent for 20 minutes and diaminobenzidine chromogen/substrate working solution for 10 minutes. Lastly, counterstaining with haematoxylin was performed.

#### Assessment of the immunostaining

Positive staining was identified as brownish nuclear staining for both SATB2 and CDX2, whereas cytoplasmic and/or membranous staining was considered for positive CK20 expression. For markers scoring, percentages of positive tumour cell were considered regardless of the staining intensity. Receiver operator characteristic (ROC) curve was plotted to identify the best SATB2 cut-off point for diagnosis of CRC. The percentage located close by the point that provides maximum sensitivity and specificity was selected as the cut-off point [12]. CDX2 and CK20 were considered positive when the percentage of stained tumour cells was 10 or more for CDX2 and 5 or more for CK20 [13, 14].

### Statistical analysis

Data were statistically analysed using the Statistical Package for the Social Sciences software version 23. Categorical variables were expressed as frequencies whereas numerical variables were expressed as mean ± SD. Accuracy, specificity, sensitivity, positive and negative predictive values (NPVs) were used to assess diagnostic values of the tested markers. The histopathologic diagnosis was considered the gold standard.

ROC curve was used to select the best cut-off point for SATB2 through assessing the diagnostic values of different percentages of SATB2 expression. Areas under the ROC curve (AUC) of each marker were calculated; higher AUC values indicate better test performance. For evaluating the level of agreement between marker expression in primary CRC and their paired metastatic specimens, concordance rate and Kappa coefficient (κ) were applied.

## Results

### Clinicopathologic characteristics of primary CRC cases

This study included 70 primary CRC cases with a mean age of 55.47 ± 12.78 years. Forty-five cases (64.3%) were male. Tumours were located in left colon in 31 cases (44%) and exhibited fungating appearance in 26 cases (37%). Conventional adenocarcinoma constituted the predominant histologic type (42 cases (60%)) and high-grade features were identified in 42 cases (60%). Vascular and perineural invasion were detected in 22 cases (31.5%) and 17 cases (24.2%), respectively. Tumours were associated with nodal involvement in 53 cases (75.8%) whereas distant metastasis was present in 24 cases (34.3%). As regard TNM staging, Stage III was the most frequent (29 cases (57%)). Clinicopathologic data of the studied cases are represented in Table 1.

### Expression of SATB2, CDX2 and CK20 in the studied primary CRC, paired metastatic CRC and non-CRC cases

**Figure 1.**
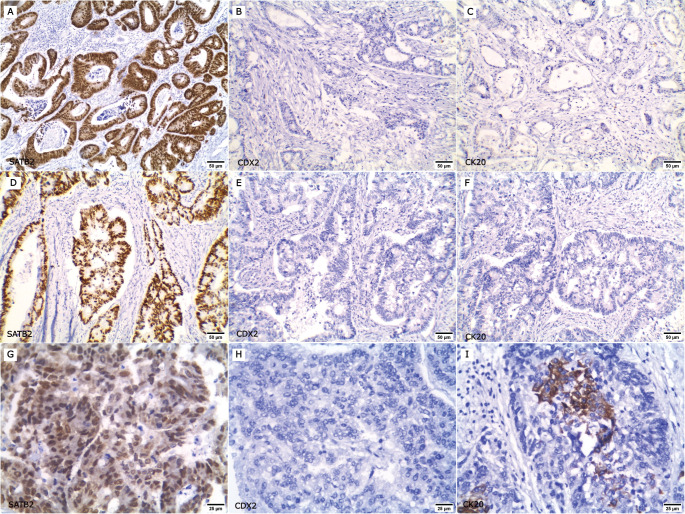
Expression of SATB2, CDX2 and CK20 in primary CRC cases. (a–f ×200): Low-grade CRC cases; (a, d): Positive for nuclear SATB2 expression, (b, e): CDX2 negative and (c, f): CK20 negative. (g–i ×400): High-grade CRC case; (g): SATB2 positive, (h): CDX2 negative and (i): CK20 focal positive.

**Figure 2.**
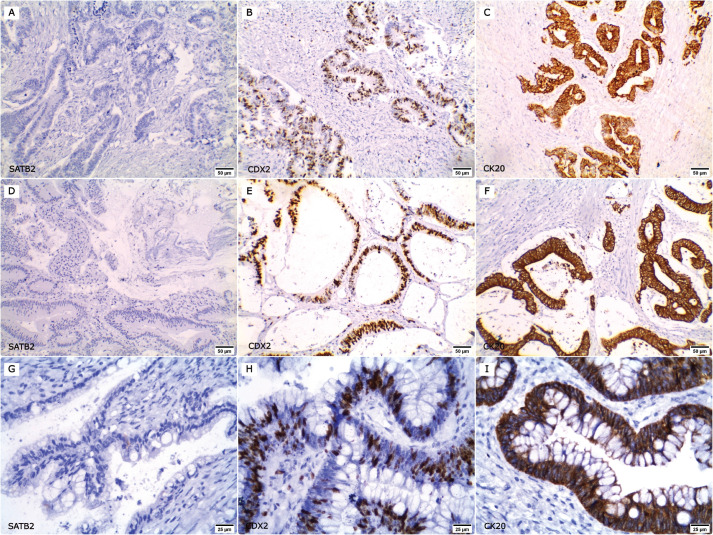
Expression of SATB2, CDX2 and CK20 in non-CRC cases. (a–c ×200): A case of urinary bladder adenocarcinoma; (a): SATB2 negative, (b): nuclear CDX2 expression and (c): membranous and cytoplasmic CK20 positivity. (d–f ×200): A case of duodenal adenocarcinoma; (d): negative for SATB2 expression whereas (e): CDX2 and (f): CK20 were positive. (g–i ×400): A case of ovarian mucinous adenocarcinoma; (g): SATB2 negative, (h): CDX2 and (i): CK20 positive.

**Figure 3.**
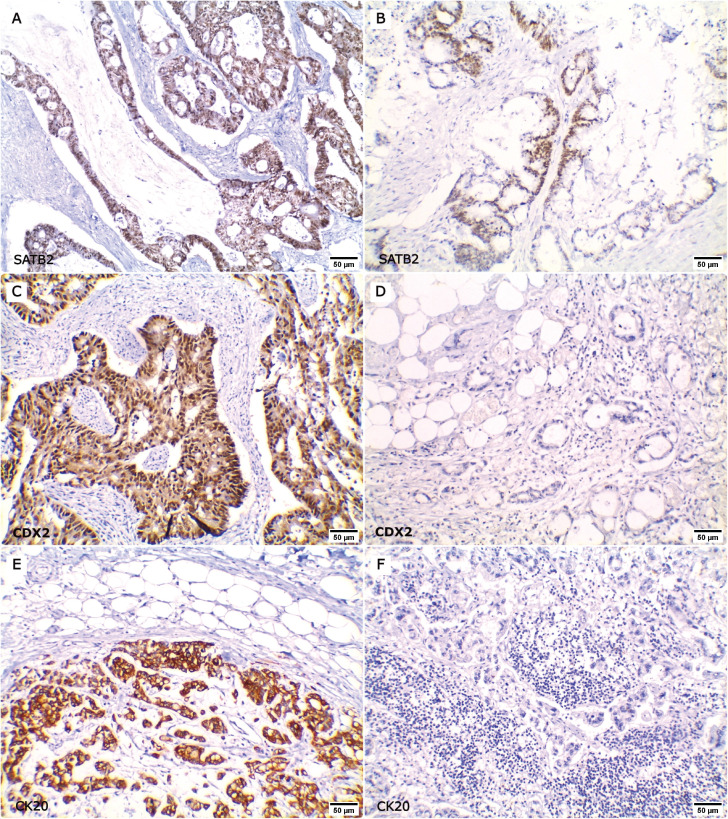
Expression of SATB2, CDX2 and CK20 in primary CRC and their paired metastatic specimens. (a–b ×200): Positive SATB2 nuclear expression in both (a): primary CRC and (b): its paired metastatic specimen. (c–d ×200): Positive CDX2 expression in (c): primary CRC and lost CDX2 positivity in (d): the metastatic site. (e–f ×200): Positive CK20 expression in (e): primary CRC and lost CK20 expression in (f): the metastatic site.

Representative figures for the expression of SATB2, CDX2 and CK20 in primary CRC, paired metastatic CRC and non-CRC cases are demonstrated in Figures 1–3. SATB2 was identified as nuclear staining in 66 (94.3%) primary CRC cases, 48 (96%) paired metastatic CRC specimens and 2 (2.5%) non-CRC cases. CDX2 nuclear expression was positive in 67 (95.7%) primary CRC cases, 46 (92%) paired metastatic CRC specimens and 10 (12.5%) non-CRC cases. Whereas CK20 was identified as cytoplasmic and/or membranous expression in 64 (91.5%) primary CRC cases, 44 (88%) paired metastatic CRC specimens and 11 (13.8%) non-CRC cases.

### Validity of single marker expression in the detection of CRC origin

ROC curve analysis of SATB2 expression alone provided 94.3% sensitivity, 97.5% specificity and 0.975 AUC (*p* < 0.001). SATB2 had 97% positive predictive value (PPV), 95% NPV and diagnostic accuracy of 96%. The ideal SATB2 cut-off value for distinguishing colonic from non-colonic cases, in this study, was 10%.

**Figure 4.**
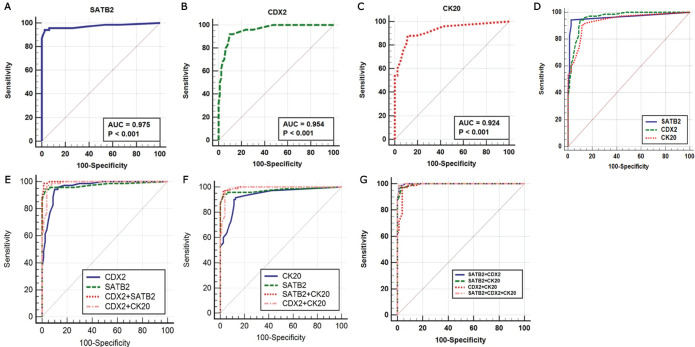
(a–g): ROC curves of single and combined markers in the diagnosis of primary CRC.

Positive CDX2 expression alone described better sensitivity (95.7%) but weaker specificity (87.5%) whereas CK20 positivity revealed the lowest sensitivity and specificity for detection of primary CRCs versus non-CRCs (sensitivity 91.4% and specificity 86.5%) as demonstrated in Table 2 and Figure 4a–d.

### Validity of combined marker expression in the detection of CRC origin

Next, we evaluated whether combinations of these three markers could improve the identification of a CRC origin. It was demonstrated that combining SATB2 and CDX2 revealed better sensitivity (98.5%), specificity (98.8%) and larger AUC (0.998) compared to SATB2 alone (94.3% sensitivity, 97.5% specificity, AUC: 0.975), CDX2 alone (95.7% sensitivity, 87.5% specificity, AUC: 0.954) and combined CDX2 and CK20 (97.1% sensitivity, 96.3% specificity, AUC: 0.987).

When combining SATB2 and CK20, the same sensitivity as combined CDX2 and CK20 (97.1%) was obtained, but it was better than that of SATB2 alone (94.3%) and CK20 alone (91.4%). The specificity of this combination was the same as SATB2 alone (97.5%) but better than CK20 alone and combined CDX2 and CK20 (86.2% and 96.3%, respectively).

Combining the three markers provided better sensitivity (98.5%) and specificity (98.8%) than combining SATB2 and CK20 (sensitivity 97.1% and specificity 97.5%) and combined CDX2 and CK20 (sensitivity 97.1% and specificity 96.3%). Whereas it had the same sensitivity and specificity of SATB2 and CDX2 combination. Both showed the highest sensitivity (98.5%) and specificity (98.8%) as illustrated in Table 2 and Figure 4e–g.

### Concordance of markers expression between primary CRCs and their paired metastatic specimens

In order to evaluate the concordance of SATB2 expression between primary and metastatic CRCs, achievable paired metastatic specimens (50 cases) were obtained. SATB2 expression was totally concordant between primary CRCs and their paired metastatic specimens (concordance rate 100% and κ coefficient = 1 denoting perfect level of agreement). As regard CDX2 and CK20, two cases lost CDX2 expression (concordance rate 96% and κ coefficient = 0.648 indicating good level of agreement) and three cases lost CK20 expression (concordance rate 94% and κ coefficient = 0.638 reflecting good level of agreement) in the paired metastatic specimens, whereas combining CDX2 and CK20 revealed concordance rate of 98% and κ coefficient of 0.790 denoting excellent level of agreement. Combining SATB2 to either CDX2 or CK20 improved the concordance (concordance rate 100% and κ coefficient = 1 denoting perfect level of agreement) as outlined in Table 3.

## Discussion

CRC metastases often simulate the histological patterns of other adenocarcinomas; differentiation between these metastases can be difficult especially in poorly differentiated adenocarcinomas [3]. There is continuous interest for identification of newer combinations of immunohistochemical markers with higher sensitivity and specificity in the diagnosis of CRC. This study investigated immunohistochemical expression of SATB2 in colorectal and non-CRCs. Also, the diagnostic value of SATB2 alone, and the double and triple combinations of SATB2 with CDX2 and/or CK20 were evaluated.

Analysing SATB2 expression in primary CRC cases, the current study demonstrated that SATB2 was detected as a brownish nuclear staining in 66 (94.3%) primary CRC specimens and only in 2 (2.5%) non-CRC specimens. SATB2 sensitivity obtained in this work (94.3%) was close to other studies in which the sensitivity ranged from 90% to 99% [3, 8, 15–18]. On the opposite side, a lower SATB2 sensitivity was noticed by other studies (71%–89%) [9, 19–23]. Regarding SATB2 specificity, this work reported a specificity of 97.5%. This was in concordance with different studies that reported SATB2 specificity between 93% and 100% [3, 17, 21, 24]. Other reports showed a lower range of specificity from 75% to 88% [4, 5, 8, 25, 26]. This wide variability in SATB2 specificity and sensitivity could be explained by differences in histological types, grade and stage of the included specimens in each study, besides the type of antibodies used. Furthermore, different types of non-CRC specimens enrolled in each study could clarify the discrepancy in SATB2 specificity.

From ROC curve analysis, in this study, the best SATB2 cut-off value that afforded the highest sensitivity and specificity for identifying the colonic origin was 10%. Lin *et al* [17] and De Michele *et al* [27] reported a lower SATB2 cut-off point at 5%. Whereas Zhang *et al* [3] considered any nuclear staining as SATB2 positive, so the cut-off point in their study was 1%. These differences may be due to different methods performed to determine cut-off values in these studies.

The cases included in this work were mostly conventional adenocarcinoma, as well as mucinous and signet ring cell carcinomas. Li *et al* [25] focused on SATB2 expression in medullary carcinoma of the large intestine. They revealed positive SATB2 expression in 89% of cases making it a promising marker for the detection of medullary colonic carcinoma.

Considering the validity of the examined markers individually, this study achieved that SATB2 was more sensitive than CK20 but less sensitive than CDX2 in detection of CRC. However, SATB2 was more specific than CDX2 and CK20 similar to the findings of other reports [3, 5, 8, 9, 23, 26, 28]. Salim *et al* [28] reported that SATB2 and CDX2 had the same sensitivity, and theirs were higher than that of CK20. In contrast to Zhang *et al* [3] who concluded that CDX2 and CK20 were more sensitive than SATB2. The discrepancies could be attributed to different types of non-CRC tumours and their histological variants. It should be mentioned that non-CRC with intestinal differentiation or of mucinous type were recognised to express high rates of CDX2 and/or CK20 opposed to SATB2 that frequently addressed negative expression in those tumours even if they show intestinal differentiation.

Combination of different markers could improve their validity in the identification of the colonic origin. Therefore, this work was extended to investigate the validity of SATB2 in combination with CDX2 and/or CK20 in the diagnosis of CRC. Combining SATB2 with CDX2 or CK20 provided better sensitivity, specificity and larger AUC compared to SATB2, CDX2 and CK20 individually.

Moreover, it was found that combining SATB2 with CDX2 provided better sensitivity, specificity and larger AUC than the commonly used combination in practice (CDX2 + CK20). It worth noting that the triple combination of SATB2, CDX2 and CK20 offered the same sensitivity, specificity and AUC as the double SATB2 and CDX2 combination. So, the current work suggested that the double combination of SATB2 and CDX2 was the best combination serving the highest sensitivity and specificity in detection of CRC.

Similar results were detected by Dabir *et al* [5] and Salim *et al* [28] who reported that combining SATB2 and CDX2 had the best sensitivity and specificity in detection of CRC. Zhang *et al* study [3] reported that combined SATB2 and CDX2 showed better specificity, but in contrast, it revealed that the traditional combination of CDX2 with CK20 had a better sensitivity than other combinations.

To the authors’ knowledge, this is the first study to analyse the level of agreement of SATB2, CDX2 and CK20, either individually or combined, between primary CRCs and their paired metastatic specimens. In the current study, SATB2 was able to retain its expression in the corresponding metastatic lesions with a perfect level of agreement as all SATB2 positive primary CRC specimens retained SATB2 expression in their metastatic specimens. Whereas either CDX2 or CK20 alone provided only a good level of agreement as CDX2 positivity was lost in two paired metastatic specimens while three metastatic specimens lost CK20 expression. Moreover, adding SATB2 to CDX2 and/or CK20 improved their ability to detect the colonic origin in the metastatic specimens.

## Conclusion

SATB2 provides high sensitivity and specificity for establishing or ruling out the diagnosis of CRC. The optimal SATB2 cut-off value that afforded the highest sensitivity and specificity for discriminating colonic from non-colonic origin is 10%. SATB2 and CDX2 is the best combination for identifying CRC. Adding SATB2 to CDX2 and/or CK20 improves their ability to detect the colonic origin in metastatic specimens. SATB2 is able to retain its expression at the metastatic sites.

## Conflicts of interest

The authors have no conflicts of interest to declare.

## Funding

The authors did not receive support from any organisation for the submitted work.

## Availability of data and material

The datasets used and/or analysed during the current study are available from the corresponding author on reasonable request.

## Author contributions

MSE: Data collection and interpretation of the histopathologic and immunohistochemical stained slides, captured the figures provided in this study, interpretation of the statistical analysis, drafting and writing the manuscript. DMG: Participated in interpretation of the histopathologic and immunohistochemical stained slides, statistical analysis and its interpretation, drafting and writing the manuscript. YAZ: Study design, participated in interpretation of the histopathologic and immunohistochemical stained slides, revision of the draft of the manuscript. AGN: Study design, supervision of the study, revision of the draft of the manuscript. FAE: Study design, supervision of the study, revision of the draft of the manuscript. All authors read and approved the final version of the manuscript.

## Ethics approval

Protocol was approved by the Research Ethics Committee in Faculty of Medicine, Tanta University (reference# 32895/01).

## Figures and Tables

**Table 1.** Patients’ characteristics.

	*N* (%)
Age (years)	
<50	22 (31.5)
≥50	48 (68.5)
Gender	
Male	45 (64.3)
Female	25 (35.7)
Tumour location	
Right colon	26 (37)
Left colon	31 (44.4)
Rectum	13 (18.6)
Tumour size (cm)	
<5	34 (48.5)
≥5	36 (51.5)
Gross features	
Fungating	26 (37)
Ulcerating	24 (34.4)
Infiltrating	20 (28.6)
Histologic type	
Conventional adenocarcinoma	42 (60)
Mucinous adenocarcinoma	24 (34.3)
Signet ring cell carcinoma	4 (5.7)
Histologic grade	
Low grade	28 (40)
High grade	42 (60)
Vascular invasion	
Present	22 (31.5)
Absent	48 (68.5)
Perineural invasion	
Present	17 (24.2)
Absent	53 (75.8)
Nodal metastasis	
Present	53 (75.8)
Absent	17 (24.2)
Distant metastasis	
Present	24 (34.3)
Absent	46 (64.7)
Staging	
I	12 (14.3)
II	5 (7.2)
III	29 (57)
IV	24 (21.5)

**Table 2.** Degree of diagnostic accuracy of single and combined markers expression for the diagnosis of CRC.

Marker	Sensitivity %	Specificity %	PPV %	NPV %	Accuracy %	AUC
SATB2 +	94.3	97.5	97	95	96	0.975
CDX2 +	95.7	87.5	87	95.8	91.3	0.954
CK20 +	91.4	86.2	85.3	92	88.7	0.924
SATB2 + / CDX2 +	98.5	98.8	98.5	98.8	98.6	0.998
SATB2 + / CK20 +	97.1	97.5	97.1	97.5	97.3	0.995
CDX2 + / CK20 +	97.1	96.3	95.8	97.5	96.6	0.987
SATB2 + / CDX2 + /CK20 +	98.5	98.8	98.5	98.8	98.6	0.998

PPV, Positive predictive value; NPV, Negative predictive value; AUC, Area under curve

**Table 3.** Concordance of single and combined markers expression between primary CRC and paired metastatic CRC specimens.

Marker	Concordance rate	kappa coefficient (κ)	Level of agreement
SATB2	100%	1	Perfect
CDX2	96%	0.648	Good
CK20	94%	0.638	Good
SATB2 and CDX2	100%	1	Perfect
SATB2 and CK20	100%	1	Perfect
CDX2 and CK20	98%	0.790	Excellent
SATB2, CDX2 and CK20	100%	1	Perfect
